# Unusual Occurrence of Two Bona-Fide CCA-Adding Enzymes in *Dictyostelium discoideum*

**DOI:** 10.3390/ijms21155210

**Published:** 2020-07-23

**Authors:** Lieselotte Erber, Anne Hoffmann, Jörg Fallmann, Monica Hagedorn, Christian Hammann, Peter F. Stadler, Heike Betat, Sonja Prohaska, Mario Mörl

**Affiliations:** 1 Institute for Biochemistry, University of Leipzig, Brüderstraße 34, 04103 Leipzig, Germany; lieselotte.erber@uni-leipzig.de (L.E.); hbetat@uni-leipzig.de (H.B.); 2 Interdisciplinary Center for Bioinformatics, Leipzig University, Härtelstraße 16-18, 04107 Leipzig, Germany; anne.hoffmann@helmholtz-muenchen.de (A.H.); fall@bioinf.uni-leipzig.de (J.F.); peter.stadler@bioinf.uni-leipzig.de (P.F.S.); sonja@bioinf.uni-leipzig.de (S.P.); 3 Ribogenetics Biochemistry Lab, Department of Life Sciences and Chemistry, Jacobs University Bremen gGmbH, Campus Ring 1, 28759 Bremen, Germany; m.hagedorn@jacobs-university.de (M.H.); C.Hammann@jacobs-university.de (C.H.); 4 German Centre for Integrative Biodiversity Research (iDiv) Halle-Jena-Leipzig, Competence Center for Scalable Data Services and Solutions, and Leipzig Research Center for Civilization Diseases, Leipzig University, 04103 Leipzig, Germany; 5 Max Planck Institute for Mathematics in the Sciences, 04103 Leipzig, Germany; 6 Facultad de Ciencias, Universidad National de Colombia, Sede Bogotá, Carrera 45 No. 26-85, Colombia; 7 Santa Fe Institute for Complex Systems, 1399 Hyde Park Road, Santa Fe, NM 87501, USA; 8 Department of Theoretical Chemistry of the University of Vienna, A-1090 Vienna, Austria; 9 Computational EvoDevo Group, Department of Computer Science, Leipzig University, Härtelstraße 16-18, 04107 Leipzig, Germany

**Keywords:** tRNA nucleotidyltransferase, CCA-adding enzyme, enzyme evolution, *Dictyostelium discoideum*

## Abstract

*Dictyostelium discoideum*, the model organism for the evolutionary supergroup of Amoebozoa, is a social amoeba that, upon starvation, undergoes transition from a unicellular to a multicellular organism. In its genome, we identified two genes encoding for tRNA nucleotidyltransferases. Such pairs of tRNA nucleotidyltransferases usually represent collaborating partial activities catalyzing CC- and A-addition to the tRNA 3′-end, respectively. In *D. discoideum*, however, both enzymes exhibit identical activities, representing bona-fide CCA-adding enzymes. Detailed characterization of the corresponding activities revealed that both enzymes seem to be essential and are regulated inversely during different developmental stages of *D. discoideum*. Intriguingly, this is the first description of two functionally equivalent CCA-adding enzymes using the same set of tRNAs and showing a similar distribution within the cell. This situation seems to be a common feature in Dictyostelia, as other members of this phylum carry similar pairs of tRNA nucleotidyltransferase genes in their genome.

## 1. Introduction

Transfer RNA (tRNA) nucleotidyltransferases belong to the polymerase β superfamily [1,2,3]. They are responsible for synthesis, maintenance and repair of the CCA sequence at the 3′-end of tRNAs [3,4], which is essential for the attachment of amino acids, recognition by elongation factors and correct positioning of the aminoacyl-tRNA in the ribosome [5,6]. Accordingly, these enzymes are described as CCA-adding enzymes. Furthermore, they are involved in tRNA quality control [7,8,9]. The synthesis of the CCA triplet is catalyzed in a template-independent but highly accurate way [5,10,11], where the enzymes discriminate ATP and CTP from GTP, UTP and dNTPs. Despite its importance for tRNA functionality and protein synthesis, the 3′-CCA end is not encoded in eukaryotic tRNA genes and has to be added posttranscriptionally, rendering the CCA-adding enzyme essential [12,13,14]. Based on the conserved tertiary structure and the composition of the catalytic core, eukaryotic CCA-adding enzymes and their bacterial counterparts belong to class II, while archaeal enzymes represent class I [3,15,16,17]. The class II catalytic core is composed of five conserved motifs A to E. While motif A is involved in the positioning of the catalytically essential magnesium ions [18,19], motifs B and D are important for discrimination against dNTPs and specific recognition of ATP and CTP, respectively [11]. Motif C is discussed as a spring element involved in the structural reorganization of the catalytic core required for high fidelity polymerization [20,21], while motif E likely contributes to the stabilization of the NTP binding pocket and might interact with the tRNA primer [11].

In 2015, a detailed bioinformatic analysis of CCA-adding enzymes in eukaryotes revealed the existence of two different subgroups of eukaryotic CCA-adding enzymes, the ancestral eukaryotic (eCCA) and the alphaproteobacterial-like (aCCA) type enzymes [22]. This analysis also unveiled many organisms with unusual occurrences of multiple genes for putative tRNA-nucleotidyltransferases and recent investigations in the model organisms *Schizosaccharomyces pombe* and *Salpingoeca rosetta* showed that the corresponding enzymes have partial activities as CC- and A-adding enzymes [23,24,25], comparable to those identified in bacteria [26,27,28,29]. Among the eukaryotic organisms carrying two tRNA nucleotidyltransferase genes in their genomes, *Dictyostelium discoideum* was also found to possess two such genes (*cca*1 and *cca*2) encoding for putative ancestral eukaryotic-type nucleotidyltransferases.

*Dictyostelia* belong to the super-group of Amoebozoa and mainly live in the upper layer of soil and litter of forests [30], where they prey on bacteria by phagocytosis [31]. A typical feature of these species is their adaptation to starving conditions by undergoing a developmental cycle that forms a multicellular organism with differentiated cell types [30,32,33]. This developmental cycle features a highly regulated program of alterations in protein expression, leading to the complex formation of a new organism [34,35,36]. Upon starvation, a cAMP signal is released, triggering aggregation and eventually the complex formation of fruiting bodies. In *D. discoideum*, this developmental cycle is completed within 24 h under laboratory conditions [37].

Here, we present the characterization of two tRNA nucleotidyltransferases encoded in the genome of *D. discoideum*. Our data show for the first time that this organism relies on the presence of two enzymes with bona-fide CCA-adding activity. Although both enzyme versions are not separated into a nuclear/cytosolic and a mitochondrial form and do not show significant differences in tRNA substrate preference within the life cycle of *D. discoideum*, they both seem to be essential. Surprisingly, gene expression data reveal an inverse expression of the corresponding *cca*1 and *cca*2 genes during the individual developmental stages. This might hint at the participation of both CCA-adding activities in the fine-tuning of the complex developmental cycle, as was shown for a remarkable number of other isoenzymes in *D. discoideum* [37,38,39,40,41,42,43].

## 2. Results

### 2.1. Dictyostelia Possess Two Genes for CCA-Adding Enzymes

**Figure 1:**
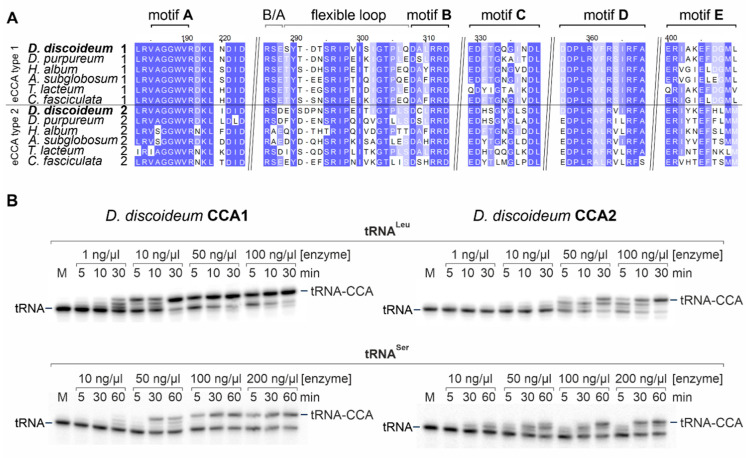
Dictyostelia species possess two CCA-adding enzymes. (**A**) Alignment of the catalytic core motifs of tRNA nucleotidyltransferases in Dictyostelia species. All enzyme sequences carry the full set of motifs required for CCA addition. Yet, in an alignment of the complete protein sequences, the enzymes can be divided into subtypes CCA1 and CCA2 and each organism carries a pair of both enzyme types. Sequences are derived from NCBI (Accession numbers: *D. discoideum* 1: XP_629100.1; 2: Q55BE1; *D. purpureum* 1: XP_003291342; 2: XP_003283778; *H. album* 1: XP_020429327; 2: XP_020431701; *A. subglobosum* 1: XP_012758000; 2: XP_012749843; *T. lacteum* 1: KYQ91040; 2: KYQ90023; *C. fasciculata* 1: XP_004361592; 2: XP_004359300). (**B**) Recombinant CCA1 and CCA2 enzymes add a complete CCA-end to radioactively labeled transcripts of *D. discoideum* tRNA^Leu^ and tRNA^Ser^. The combined time and enzyme concentration series indicate that CCA1 is faster and more efficient in the reaction than CCA2. For both enzymes, tRNA^Leu^ seems to represent a better substrate than tRNA^Ser^, as it requires less enzyme concentration and incubation time to add a CCA-end, while for tRNA^Ser^, higher concentrations are required. Yet, both tRNA substrates are accepted by CCA1 as well as CCA2, indicating that the substrate itself does not affect the enzymes. M, mock incubation of labeled tRNA transcripts lacking the CCA-terminus; enzymes were added at indicated final concentrations. Incubation times are given in minutes (min).

Recently, the existence of two genes *cca*1 and *cca*2 encoding for putative tRNA nucleotidyltransferases in *Dictyostelium discoideum* was described [22,44]. To clarify whether this situation represents a common feature in the group of Dictyostelia, we analyzed annotated genomes of *Acytostelium subglobosum*, *Cavenderia fasciculata*, *Dictyostelium discoideum*, *Dictyostelium purpureum*, *Heterostelium album* and *Tieghemostelium lacteum*. In all these species, we identified two genes encoding for putative CCA-adding enzymes with a high conservation of the catalytic core motifs (Figure 1A). Furthermore, all sequences were identified to represent the ancestral eukaryotic type of CCA-adding enzymes (eCCA). According to our splitstree analysis, each pair of enzyme sequences can be divided into subgroups eCCA type 1 and eCCA type 2 (Figure S1). Members of type 1 share higher sequence similarity/identity, whereas members of type 2 are more heterogeneous (e.g., *D. discoideum* CCA1 and CCA2: identity: 28.4%; similarity: 44.7%). Furthermore, the *D. discoideum* CCA2 enzyme shows an enrichment of short poly-glutamine (Q)- and poly-asparagine (N)-stretches as described for many *D. discoideum* proteins [45]. The phylogenetic tree depicted in Figure S1 offers interesting insights into the evolution and origin of the two tRNA nucleotidyltransferase genes in Dictyostelia and closely related Entamoebozoa, in which also two genes for putative tRNA nucleotidyltransferases are found in the genome. As the two Entamoebozoa subtrees resemble the species phylogeny, the occurrence of two genes is very likely the result of a gene duplication event. In contrast, the distinct branches of Dictyostelia tRNA nucleotidyltransferase types exclude a recent gene duplication and hint to a horizontal gene transfer event (HGT) in the common ancestor.

### 2.2. Both Enzymes of D. discoideum Are Fully Active CCA-Adding Enzymes

To characterize the catalytic properties of both tRNA nucleotidyltransferases in *D. discoideum*, the coding regions of *cca1* (DDB_G0271378, chromosome 2; coordinates 147174 to 148936) and *cca2* (DDB_G0293504, chromosome 6; coordinates 2968920 to 2970687) were cloned as codon-optimized sequences (GenScript) for recombinant expression in *E. coli*. Purified enzymes CCA1 and CCA2 were tested for activity on radioactively labeled in vitro transcripts of *D. discoideum* tRNA^Leu^_(UAA)_ and tRNA^Ser^_(GCU)_. Reaction products were separated on denaturing polyacrylamide gels and visualized by autoradiography (Figure 1B). While both tRNAs are accepted as substrates, the CCA addition is more efficient on tRNA^Leu^ than on tRNA^Ser^, where higher enzyme concentrations and longer incubation times are required for both CCA1 and CCA2. An explanation for this discrepancy in substrate efficiency is probably the fact that tRNA^Leu^ carries an A residue at the discriminator position 73, immediately upstream of the CCA-end, while tRNA^Ser^ carries a G at the corresponding position. It is known that A73 is the preferred 3′-end for CCA addition, while other residues are accepted to a lesser extent [46]. Furthermore, it is possible that lacking modifications affect the folding of the in vitro transcribed tRNAs to different extents. While the results indicate that both enzymes have similar substrate preferences, the analysis shows that CCA1 and CCA2 differ in their overall efficiency of polymerization. On both tRNA substrates, CCA1 is already active at low concentrations (tRNA^Leu^: 1 ng/µL at 30 min; tRNA^Ser^: 50 ng/µL at 30 min) and incubation times. In contrast, CCA2 shows efficient CCA addition only at higher concentrations and increased incubation times (tRNA^Leu^: 50 ng/µL at 30 min; tRNA^Ser^: 100 ng/µL at 60 min). Yet, these analyses clearly show that both enzymes exhibit a bona fide CCA-adding activity on different tRNA substrates (Figure 1A, Figure S2). This is further supported by sequence alignments with eCCA-type enzymes of known activity, where all motifs required for full CCA addition were identified in CCA1 and CCA2 (Figure S3).

### 2.3. A Knock-Out of CCA1 and CCA2 Was not Successful, and Both Gene Products Show Similar Localization Patterns

CCA1 and CCA2 show identical activities, raising the possibility that they represent compensating back-up activities as described for the functional redundancy of tRNA 3′-end processing endo- and exonucleases in Bacteria [47,48]. To address this question, several attempts based on homologous recombination [49] and CRISPR-Cas9 activity [50] to knock-out the individual genes were performed in the laboratory strain *D. discoideum* Ax2 [51]. Yet, no cells carrying gene disruptions of *cca*1 or *cca*2 could be obtained, indicating that both genes do not complement each other.

**Figure 2:**
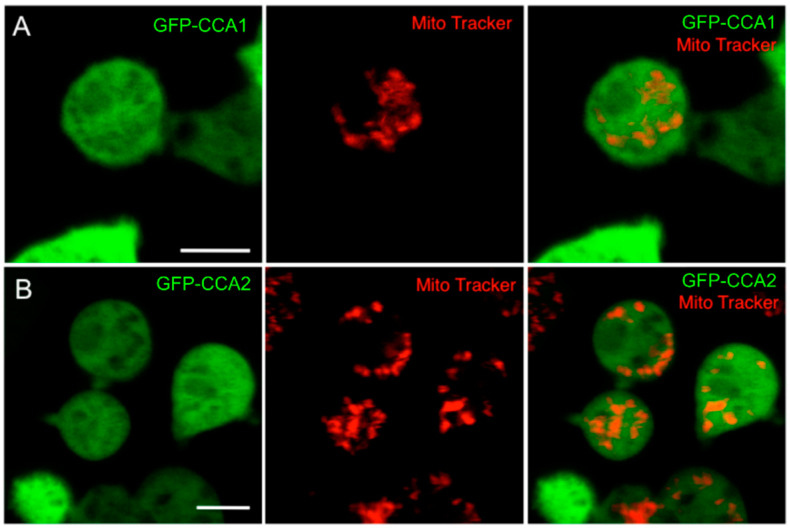
Subcellular localization of CCA1 and CCA2 enzymes. Full length CCA1 and CCA2 enzymes fused to GFP ((**A**): GFP-CCA1; (**B**): GFP-CCA2) were overexpressed in wild-type *Dictyostelium* cells and show a cytosolic distribution but no specific enrichment within mitochondria (red, stained with MitoTrackerRed). Size bar represents 5 µm.

A possible explanation for the absence of a functional redundancy is that one of the enzymes is responsible for CCA addition on nuclear encoded cytosolic tRNAs, while the second enzyme acts on the mitochondrial tRNA pool. Hence, the subcellular localization of CCA1 and CCA2 was investigated (Figure 2). The coding sequences of CCA1 and CCA2 were inserted into the *Dictyostelium* expression plasmid pDM317, resulting in the constitutive expression of the corresponding proteins with an N-terminal GFP tag, a standard approach to monitor subcellular protein localization in *D. discoideum*. Mitochondria were stained with MitoTrackerRed (CMXRos) and cells were documented by live fluorescence imaging. Both tagged enzymes showed similar localization patterns with an even distribution in the cytosol and were not specifically enriched in mitochondria.

### 2.4. Expression of CCA1 and CCA2 is Cell-Cycle-Dependent and Inversely Regulated

**Figure 3:**
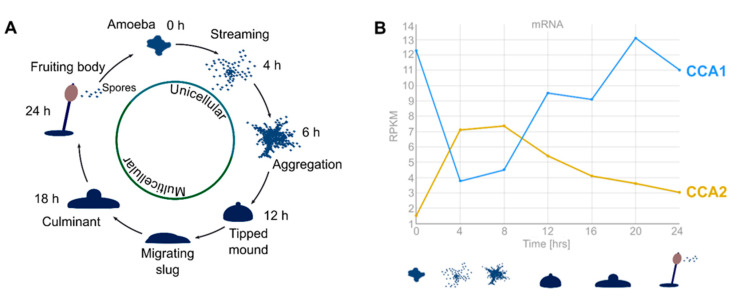
CCA1 and CCA2 are oppositely regulated during *D. discoideum* development. (**A**) Schematic presentation of the *D. discoideum* life cycle. Upon starvation, cells release cAMP as an external signal and move towards an aggregation center, where they form a multicellular organism. Eventually, this organism forms a fruiting body consisting of differentiated spore and stalk cells. Under improved growth conditions, the released spores grow out again into vegetative cells. (**B**) mRNA profiles of *cca1* (DDB_G0293504) and *cca2* (DDB_G0271378) genes according to dictyExpress. At time point 0, mRNA levels of *cca*1 are high and decrease during the first hours of development, increase again after 8 h and reach the initial level during the final stages of development. In contrast, mRNA levels of *cca2* are close to zero at time point 0 and increase to a maximum after 4 to 8 h. Then, the levels decrease continuously until 24 h. Taken together, the diagram shows a diametrically contrary transcription of *cca1* and *cca2* genes, indicating specific functions during the life cycle.

While both enzymes have identical catalytic activities and overlapping cellular localizations, we addressed the question as to whether *cca1* and *cca2* exhibit a different cell-cycle-correlated temporal expression (Figure 3). Expression profiles were examined using the dictyExpress database, which provides information on mRNA levels of *Dictyostelium* spp. at different time points during the developmental cycle [52]. For the analysis, RNA-seq data from Parikh et al. were used [34]. In the unicellular state, *cca1* is highly expressed. Immediately after the onset of aggregation (representing the start of the cellular differentiation process), transcription of *cca1* is downregulated (4 to 8 h) and raises again when the cells switch from aggregation to mound formation until the final fruiting body is formed (12 to 24 h) (Figure 3B). Surprisingly, *cca2* exhibits a perfect opposite expression profile. With a very low expression in amoeba, *cca2* is upregulated as soon as the developmental process into the aggregation is started (4 to 8 h) and declines for the rest of the development (12 to 24 h). Hence, mRNA expression of *cca1* and *cca2* is exactly oppositely regulated in a coordinated and strictly development-dependent way. Taken together, these expression profiles result in a constant level of CCA-adding activity composed of different quantities of CCA1 and CCA2 in *D. discoideum* over the whole life cycle.

### 2.5. CCA1 and CCA2 Have Identical Substrate Specificities

**Figure 4:**
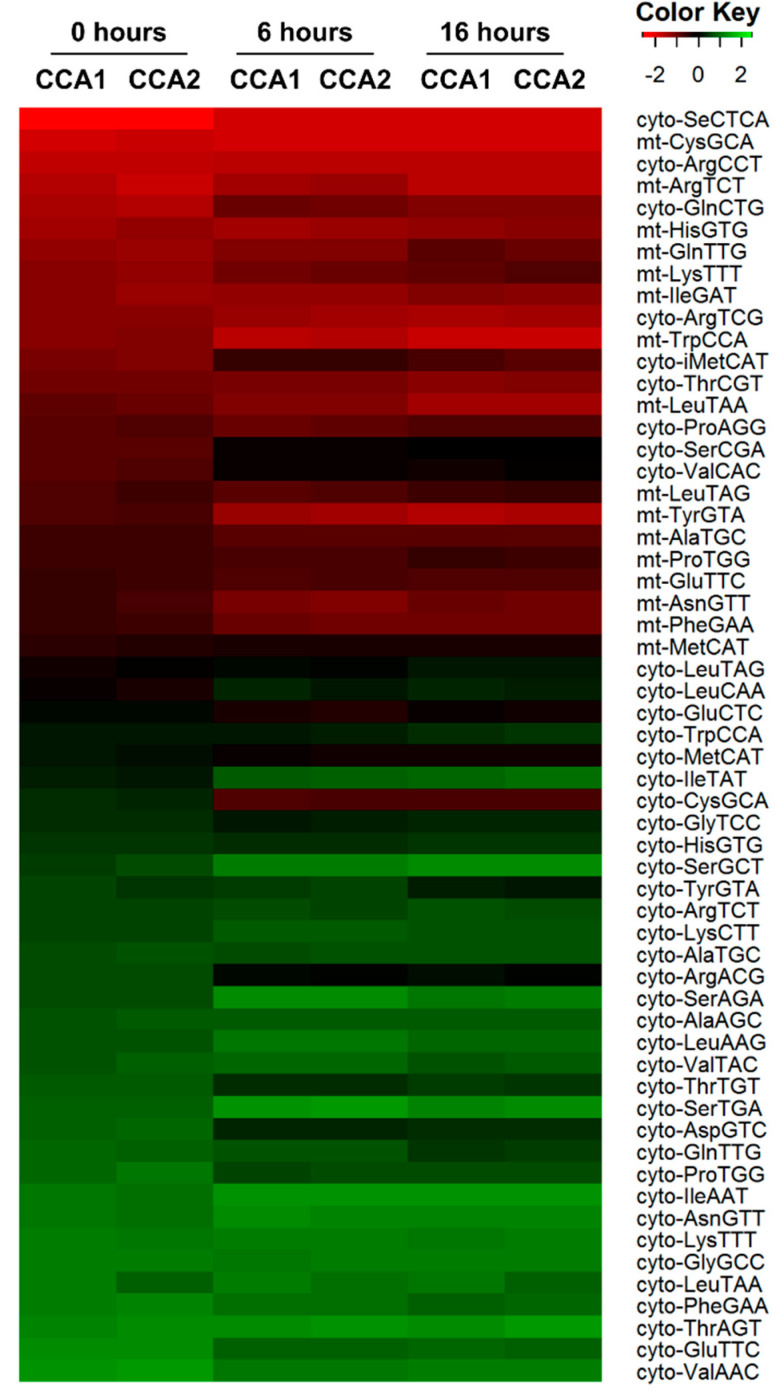
Substrate specificity of CCA1 and CCA2 at different developmental time points. CCA-depleted tRNAs of *D. discoideum* in different developmental stages (0 h, 6 h and 16 h) were incubated with recombinant CCA1 or CCA2 for CCA addition. Reaction products were analyzed by LOTTE-seq [54]. Data were visualized as heatmap using the program R statistic. No significant differences in substrate selection by CCA1 and CCA2 were visible, indicating that both enzymes have very similar substrate specificities.

To investigate in detail whether the two CCA-adding enzymes of *D. discoideum* differ in their selectivity for certain tRNAs as substrates for CCA incorporation, an in vitro specificity test was established and combined with high-throughput sequencing. Because of the inversely regulated transcription of *cca*1 and *cca*2, tRNA samples from different time points during development were isolated (0 h, 6 h and 16 h after development onset). Subsequently, 3′-CCA ends of the tRNA pools were removed by snake venom phosphodiesterase I (SVPD) treatment, an exonuclease that can be easily adjusted to remove only two to three nucleotides from the 3′-end of tRNAs [53,54]. The SVPD-treated tRNA pools were offered as substrates for CCA addition by CCA1 and CCA2, respectively. To investigate a possible substrate specificity, it is essential to perform the in vitro CCA incorporation under non-saturating conditions and we determined the required amount of each enzyme beforehand. Libraries of the reaction products were prepared according to the LOTTE-seq protocol [54], resulting in more than 3 million tRNA reads with >99% ending in CCA. As a result, we found only minor differences in the tRNA identity of CCA1 and CCA2 reaction products, suggesting that both enzymes accept the complete sets of cytosolic and mitochondrial tRNAs at the individual developmental stages (Figure 4).

### 2.6. CCA1 and CCA2 Differ in Their Affinities for tRNA Substrates

**Figure 5:**
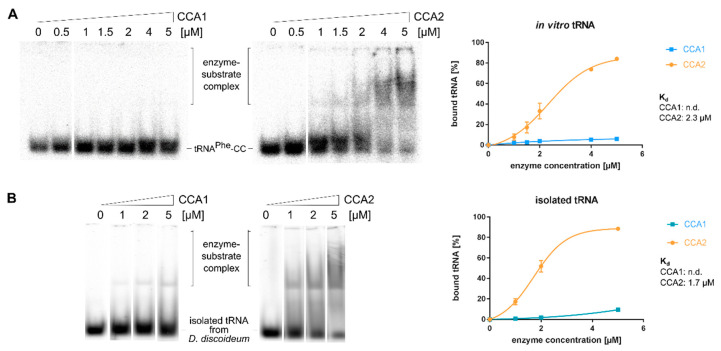
Distinct binding affinities of CCA1 and CCA2. Electrophoretic mobility shifts of CCA1 and CCA2 with in vitro transcribed yeast tRNA-CC (**A**) or total in vivo tRNA of *D. discoideum* (**B**). Left panel shows representative gel shifts. While CCA1 showed no binding over the whole concentration range (0–5 µM), CCA2 efficiently interacted with its substrates, resulting in clear shift signals. Signals of electrophoretic mobility shift assays (EMSA) were densitometrically quantified and used for determination of binding constants (right panel). For CCA2, binding constants of 1.7 µM (in vivo tRNA) and 2.3 µM (in vitro tRNA) were determined, while CCA1 interaction is too weak to allow for a calculation of its substrate affinity.

Interestingly, the genome of *D. discoideum* encodes for an unusually high amount of Q/N-rich proteins carrying stretches of poly-glutamine and poly-asparagine [45]. As it is discussed that these stretches are specifically linked to RNA-binding domains [45], it is conceivable that the Q/N-rich CCA2 enzyme differs in its affinity for tRNA substrates from its Q/N-lacking counterpart CCA1. Hence, the tRNA binding behavior of both enzymes was tested by electrophoretic mobility shift assays (EMSA). Radioactively labeled in vitro transcribed, as well as isolated in vivo tRNA substrates ending with CC, were incubated with increasing amounts of recombinantly expressed CCA1 or CCA2 enzyme and separated by native polyacrylamide gel electrophoresis (Figure 5A). Enzyme-bound and free substrates were visualized and quantified on a phosphor imager device. CCA2 showed efficient binding to the substrates with a K_d_ of 2.3 µM and 1.7 µM for the in vitro transcript and the in vivo tRNA preparation, respectively (Figure 5B). In contrast, CCA1 showed almost no binding at any protein concentration. Nevertheless, both enzyme preparations represent functional proteins showing efficient and comparable CCA addition in vitro (Figure S3). In addition, a preincubation of the enzymes with 1mM non-hydrolysable ATPγS had no effect on the efficiency of their tRNA binding, excluding any allosteric effects on the substrate affinity (Figure S4). Furthermore, when incubated with CTP or ATP alone, both enzymes showed an almost identical binding behavior, excluding differences in their nucleotide affinities (Figure S5).

Taken together, these data clearly demonstrate that the main difference between CCA1 and CCA2 is the increased tRNA substrate affinity and lower catalytic activity of CCA2 compared to CCA1.

## 3. Discussion

While most organisms rely on the existence of a single CCA-adding enzyme for synthesis and maintenance of functional tRNA 3′-ends, many bacteria are known that carry two collaborating enzymes with partial activities for CC- and A-addition [26,27,28,55,56]. Recently, eukaryotes with split CC- and A-adding activities have also been described [23,24,25]. Hence, it is very surprising that *D. discoideum* encodes a pair of two bona-fide CCA-adding enzymes (CCA1 and CCA2) with redundant activities where a single enzyme would be sufficient. A very recent gene duplication resulting in the simultaneous expression of identical activities can be excluded, as all investigated species of the phylum Dictyostelia, including the basal *Cavenderia fasciculata* [57], carry two genes for CCA-adding enzymes. We therefore hypothesize that the last common ancestor of Dictyostelia (~600–100 mya [58]) already possessed these two genes for CCA addition. Furthermore, the distinct branches of Dictyostelia CCA1 and CCA2 in the phylogenetic analysis (Figure S1), as well as the low sequence conservation between these enzyme types, exclude a gene duplication event, although both gene versions are of eukaryotic origin [22]. Rather, the two genes presumably result from a horizontal gene transfer (HGT) in the common ancestor of Dictyostelia. Interestingly, CCA2 is characterized by a significant number of poly-glutamine (Q) and poly-asparagine (N) stretches—a typical feature of many proteins of *D. discoideum* [45,59,60]. We speculate that CCA2 might correspond to the ancestral CCA-adding enzyme of *Dictyostelium discoideum*, whereas CCA1 was acquired by a HGT. This hypothesis is strengthened by the fact that the CCA1 enzymes of Dictyostelia are more closely related to each other than the CCA2 enzymes, indicating an earlier evolutionary separation of the latter.

The observation that neither gene could be knocked-out in the *D. discoideum* amoeba indicates that both CCA1 and CCA2 likely represent essential activities. As the lethal effect of a gene knock-out in this obligate haploid organism is difficult to prove, the impossibility to obtain knock-out mutants for *cca*1 and *cca*2 might be due to technical reasons. Still, it is very likely that both *cca*1 and *cca*2 genes are indispensable and do not complement each other in their function. This is further supported by the fact that the *Dictyostelium* database does not list knock-out mutants for CCA1 or CCA2 [61]. Obviously, both enzymes are required for cell functionality and cannot complement each other. A possible reason could be that CCA1 and CCA2 fulfill different tasks in the cellular compartments since cytoplasmic as well as mitochondrial tRNAs do not encode the CCA end [62]. Computational predictions of mitochondrial target sequences is not meaningful in this case, as the *Dictyostelium* mt import system is not well understood [63]. Furthermore, as the GFP fusions of CCA1 and CCA2 are both evenly distributed in the respective cell compartments, a different substrate specificity in terms of cytoplasmic and mitochondrial tRNAs is unlikely (Figure 2). This hypothesis is corroborated by our tRNA-seq data, where no significant differences in the substrate specificity of CCA1 or CCA2 were detected, indicating that both enzymes are equally able to process mitochondrial as well as cytosolic tRNA pools throughout the complete life cycle (Figure 4).

A prominent difference between CCA1 and CCA2 is the presence of poly-Q/N stretches in CCA2 (14% asparagine and 5% glutamine). In general, *D. discoideum* open reading frames carry a high portion of CAA- (glutamine) and AAC-rich (arginine) sequences [64]. Accordingly, several hundred *D. discoideum* proteins carry long homopolymeric tracts of glutamine and/or asparagine [58,59,60,65,66]. This prevalence implies that such poly-Q/N stretches might have biological functions in *D. discoideum* [45]. As poly-Q tracts tend to aggregate and are responsible for several neurodegenerative diseases in humans, the high percentage of poly-Q-containing proteins indicates that *D. discoideum* developed mechanisms to compensate for their detrimental effects [67]. In fact, there are a large number of chaperones that obviously have coevolved to prevent aggregation of poly-glutamine-containing proteins in the cell [60,66]. Several recent studies have revealed that many Q/N-rich proteins are significantly enriched during multicellular development and are involved in the regulation of developmental processes in *D. discoideum* [59,60,64,68]. In the inverse expression profiles of CCA1 and CCA2 during the life cycle of *D. discoideum*, mRNA levels of Q/N-rich CCA2 are upregulated during cell aggregation (Figure 3B), indicating that this enzyme has a specific function at this developmental stage. Based on dictyExpress data, such differentiation correlated with changes in gene expression profiles are also observed for several RNases [41] and enzymes involved in tRNA modification (methyltransferase) [69].

Furthermore, the differential expression of isoenzyme forms is also described for a large number of *D. discoideum* proteins with possible regulatory potential such as ribonuclease [41], protein kinases [40], protein tyrosine phosphatase PTP3 [43], DNA helicase [70], chromatin remodeler CHD [71] and tRNA^His^ guanylyltransferases (Thg1) [72] in *D. discoideum*. For most of these isozymes, different functions and substrates could be identified. Taken together, this differentiation-dependent regulation seems to represent a general feature during the onset of multicellularity and cell differentiation for accurate development of the vegetative amoeba into a fruiting body [32,34,36,73,74].

A second significant difference between CCA1 and CCA2 is the surprisingly high substrate affinity of CCA2. Due to their inefficient tRNA binding, no binding constants for eukaryotic or bacterial CCA-adding enzymes are described [21,29,75]. While CCA1 shows a comparable low binding behavior, CCA2 exhibits a rather efficient interaction with native as well as in vitro transcribed tRNAs (Figure 5). It is possible that the Q/N tracts of CCA2 contribute to this increased tRNA affinity, as the positive charge of the corresponding side chains in Q/N regions might facilitate efficient binding to the negatively charged tRNA. The structure prediction for CCA2 shows that such a contribution to tRNA binding is indeed conceivable, as two Q/N tracts are in close vicinity to the catalytic core of the enzyme, where the tRNA primer is bound (Supplementary Figure S6). This scenario is corroborated by the observation that poly-Q/N stretches are frequently found in proteins which contain RNA-binding domains [45,59,65,76,77].

Interestingly, the high substrate affinity of CCA2 does not lead to a higher catalytic activity. In contrast, CCA addition is less efficient compared to CCA1, which has a low tRNA affinity. Yet, it is known for class II CCA-adding enzymes that the rate-limiting step in catalysis is indeed product release [78,79,80]. This explains why class II CCA-adding enzymes usually show a low tRNA affinity, as it is observed for CCA1 (Figure 5) [21,29,75]. Consequently, the increased tRNA binding of CCA2 seems to interfere with high catalytic activity and reduces the efficiency in CCA addition (Figure 1). Yet, both enzymes add a CCA-end to all tRNA molecules, indicating identical substrate requirements. While the Q/N tracts of CCA2 might be involved in tight tRNA binding and be responsible for a limited reaction efficiency, it is possible that they have a function completely unrelated to catalysis. In *D. discoideum*, ammonia is released by protein catabolism [81] and acts as a morphogen, affecting a number of pivotal steps during multicellular development [82,83,84]. High concentrations of ammonia are toxic for the cell and the production of glutamine and, to a lesser extent, also asparagine, is important for its detoxification, as these amino acids act as sinks for ammonia [42,85]. Indeed, glutamine synthetase is developmentally regulated in *D. discoideum* and affects growth and differentiation [86,87]. Hence, the stretches consisting of glutamine and asparagine in CCA2 may contribute to the regulation of ammonia level during the *D. discoideum* life cycle. The Q/N tracts might have a storage function for these amino acids, when glutamine and asparagine are produced at high levels by the corresponding synthetases. Alternatively, proteins with Q/N stretches like CCA2 might represent a morphogen reservoir, and their regulated degradation could be used to release ammonia for morphogenesis.

Such differences in function would represent an explanation for the existence of two CCA-adding enzymes in Dictyostelia. Yet, before the uptake of the *cca*1 gene by a HGT in the common ancestor, the single CCA2 enzyme must have been sufficient for cell survival. A prerequisite for this is a permanent expression of the *cca2* gene in order to allow the synthesis of functional tRNA molecules over the whole life cycle. After the uptake of *cca1*, the endogenous *cca2* was not replaced, and the temporal order of *cca1* and *cca2* expression could evolve, where the putative additional functions of the endogenous CCA2 enzyme are only required at certain developmental stages, while the sole CCA addition in other stages is supplied by the activity of the acquired CCA1 enzyme.

## 4. Material and Methods

### 4.1. Isolation of D. discoideum Total RNA and tRNA

Total RNA was isolated from *D. discoideum* Ax2 cells of different developmental stages (0, 6, 16, 20 and 24 h after starvation) with TRIzol^®^ (Thermo Scientific, Waltham, MA, USA) according to the manufacturer’s instructions. Small RNAs were isolated according to Carra et al. for separation of low and high molecular weight RNAs [88]. Briefly, NaCl (final concentration 0.5 M) and PEG8000 (final concentration 5%) were added to total RNA and incubated for 30 min at −20 °C. After centrifugation for 30 min at 4 °C and 10,000× *g*, the procedure was repeated with the supernatant. Small RNAs were precipitated with ethanol (100%) and separated by denaturing polyacrylamide gel electrophoresis. Bands were visualized by UV shadowing and molecules in a size range corresponding to tRNAs (60–100 nts) were extracted from the gel using the crush and soak method [89].

### 4.2. Degradation of the 3′-CCA-end of tRNAs with Snake Venom Phosphodiesterase I

A total of 500 µU snake venom phosphodiesterase I (Sigma Aldrich, St. Louis, MO, USA) was incubated with 17 µg tRNA preparation. The reaction was carried out in 400 mM Tris-HCl (pH 8.4) and 100 mM MgCl_2_ for 1 h at 25 °C. EDTA (5 mM) was added to stop the reaction. Reaction products were purified by phenol-chloroform extraction and ethanol precipitation [90].

### 4.3. Expression and Purification of D. discoideum tRNA Nucleotidyltransferases

As the genes of *D. discoideum* are exceptionally AT-rich, the ORFs of both *cca1* and *cca2* were codon optimized for overexpression in *E. coli.* Corresponding sequences with an encoded C-terminal His tag in pET28b (+) vector were purchased from GenScript (Piscataway, NJ, USA). Recombinant enzymes were expressed in *E. coli* BL21 DE3 cells with a knock-out of the endogenous tRNA nucleotidyltransferase gene (*cca*:cam; Δ*cca*) and purified as described [91]. Protein fractions were stored in 10% glycerol at −80 °C until use.

### 4.4. In Vitro Activity Test of tRNA Nucleotidyltransferases

Internally labeled tRNAs were in vitro transcribed from cloned tRNA sequences as described [92,93]. Varying amounts of CCA1 or CCA2 enzyme were incubated with 5 pmol tRNA and a mix of 1 mM NTPs (equimolar amounts of ATP, CTP, GTP and UTP) for indicated times at 20 °C in 30 mM HEPES/KOH (pH 7.6), 30 mM KCl, 2 mM DTT and 6 mM MgCl_2_. Reaction products were ethanol precipitated, separated by denaturing polyacrylamide gel electrophoresis and visualized on a Typhoon 9410 Scanner (GE Healthcare, Chicago, IL, USA). For high-throughput sequencing analysis, up to ~500 pmol of phosphodiesterase-treated tRNA of *D. discoideum* was used with adjusted limited amounts of enzyme to avoid saturating conditions.

### 4.5. High-Throughput Sequencing of tRNAs with 3′-CCA End

tRNAs treated with snake venom phosphodiesterase and CCA1 or CCA2 were analyzed according to LOTTE-Seq [54] with the exception that TGIRT (thermostable group II intron reverse transcriptase) was used for reverse transcription due to an increased yield of full-length cDNA.

### 4.6. Electrophoretic Mobility Shift Assay (EMSA)

For electrophoretic mobility shift assays 0.5 pmol α^32^P-ATP labeled tRNA^Phe^ with 3′-CC end or isolated tRNA of *D. discoideum* was used in three independent experiments in the presence or absence of 1mM non-hydrolysable ATPγS. For visualization of the in vivo tRNA pool, the 3′-CCA end was removed by snake venom phosphodiesterase as described and incubated with CCA1 and CCA2 in the presence of α^32^P-CTP to produce tRNA with a radioactively labeled 3′-CC end. tRNA was purified by phenol-chloroform extraction and ethanol precipitation [90]. tRNA was heated for 2 min at 90 °C (in vivo tRNA: 65 °C) and incubated with 0 to 5 µM enzyme in HEPES/KOH (pH 7.6), 30 mM KCl and 6 mM MgCl_2_ at 20 °C for 10 min. After addition of 80% glycerol (final concentration: 18.5%), tRNAs were separated on a 5% native PAA gel. For visualization, a Typhoon 9410 scanner was used (GE Healthcare).

### 4.7. Localization of the CCA-Adding Enzymes in Amoebozoal D. discoideum by GFP-Labeling

Coding sequences of *Dictyostelium* CCA1 (DDB_G0293504) and CCA2 (DDB_G0271378) were cloned into the *Dictyostelium* expression plasmid pDM317 [94]. After transformation of *Dictyostelium* wild-type cells (Ax2), cells expressing CCA1 and CCA2 with an N-terminal GFP were selected using G418 (10 µg/ml) in *Dictyostelium* growth medium (HL5). For imaging the intracellular localization of the CCA fusion constructs, cells were plated in µ-dishes (ibidi) and loaded with MitoTrackerRed CMXRos (ThermoFisher, Waltham, MA, USA) for 30 min (final concentration 200 nM). Live cells were imaged using a Zeiss confocal microscope.

### 4.8. Gene Deletion by Homologous Recombination

To replace the coding region for CCA1 or CCA2 by a blasticidin resistance cassette, corresponding knock-out plasmids were generated based on plasmid pKOSG-IBA (IBA-Lifesciences, Göttingen, Germany) as described [49]. Briefly, approximately 500 bp fragments of the 5′- and 3′-ends of the coding sequences were amplified from the genome and inserted into the plasmid, making up the right-arm (RA) and left-arm (LA) flanking the blasticidin cassette. The plasmid was introduced into *Dictyostelium* wild-type cells (Ax2) and blasticidin was added to the growth medium as selection pressure at a concentration of 10µg/ml.

### 4.9. Phylogenetic Analysis by Splitstree Network

Alignments of putative or verified sequences of the two tRNA-nucleotidyltransferases from the Dictyostelia species *Acytostelium subglobosum*, *Cavenderia fasciculata*, *Dictyostelium discoideum*, *Dictyostelium purpureum*, *Heterostelium album* and *Tieghemostelium lacteum* were computed using Clustal Omega [95]. Phylogenetic networks were constructed with the program SplitsTree4 (version 4.14.8, built 15 Nov 2018) using standard parameter settings [96].

### 4.10. Bioinformatic Analysis of the High-Throughput Sequencing Data

tRNAs were annotated with tRNAscan-SE v2.0 [97] using the default model for eukaryotes. To fit tRNAs to the standard tRNA model, annotated tRNA sequences were aligned against the tRNAdb database [13] containing the missing secondary structure notation. BLAST v2.4.0 [98] was used for sequence alignment and only those tRNA database entries were selected that showed the closest evolutionary similarity to the annotated tRNAs. Due to the high evolutionary distance of some tRNAs to the database entries, the notation of some tRNAs had to be adjusted manually based on the base pair rules. Mapping and read filtering of the high-throughput data were performed as described previously [54]. The genome of *D. discoideum* (dicty 2.7, assembly GCA_000004695) was downloaded from NCBI (Database resources of the National Center for Biotechnology Information 2016).

## 5. Conclusions

We have identified the first organism with two tRNA nucleotidyltransferases that both synthesize complete CCA termini without an evident tRNA substrate specificity. Our data indicate that the redundant activities of both CCA1 and CCA2 in *D. discoideum* seem to be essential throughout the individual developmental stages. Whether these enzymes, in particular CCA2, exhibit an additional function besides tRNA maturation, is a fascinating aspect and deserves further investigations.

## Supplementary Materials

Supplementary materials can be found at https://www.mdpi.com/1422-0067/21/15/5210/s1. Figure S1. Phylogenetic analysis of the two Dictyostelia CCA-adding enzyme types. Figure S2. Both CCA1 and CCA2 synthesize full CCA-ends. Figure S3. Sequence alignment of the two D. discoideum CCA-adding enzymes with eukaryotic CCA-adding enzymes with known CCA-adding activity. Figure S4. Interaction of CCA1 and CCA2 with tRNA-CC is not affected by ATP. Figure S5. CTP and ATP binding behavior of CCA1 and CCA2. Figure S6. Structure prediction of the two D. discoideum CCA-adding enzymes.

## Abbreviations

*cca1*	gene encoding enzyme CCA1
*cca2*	gene encoding enzyme CCA2
GFP	green fluorescent protein
HGT	horizontal gene transfer
ORF	open reading frame
