# Supplementary material for: Unusual Occurrence of Two Bona-Fide CCA-Adding Enzymes in Dictyostelium discoideum
Source: Int J Mol Sci. 2020 Jul 23;21(15):5210. doi: 10.3390/ijms21155210 (PMC7432833; doi:10.3390/ijms21155210)
Supplement: Supplementary file 1 [file ijms-21-05210-s001.pdf]

Supplementary Data

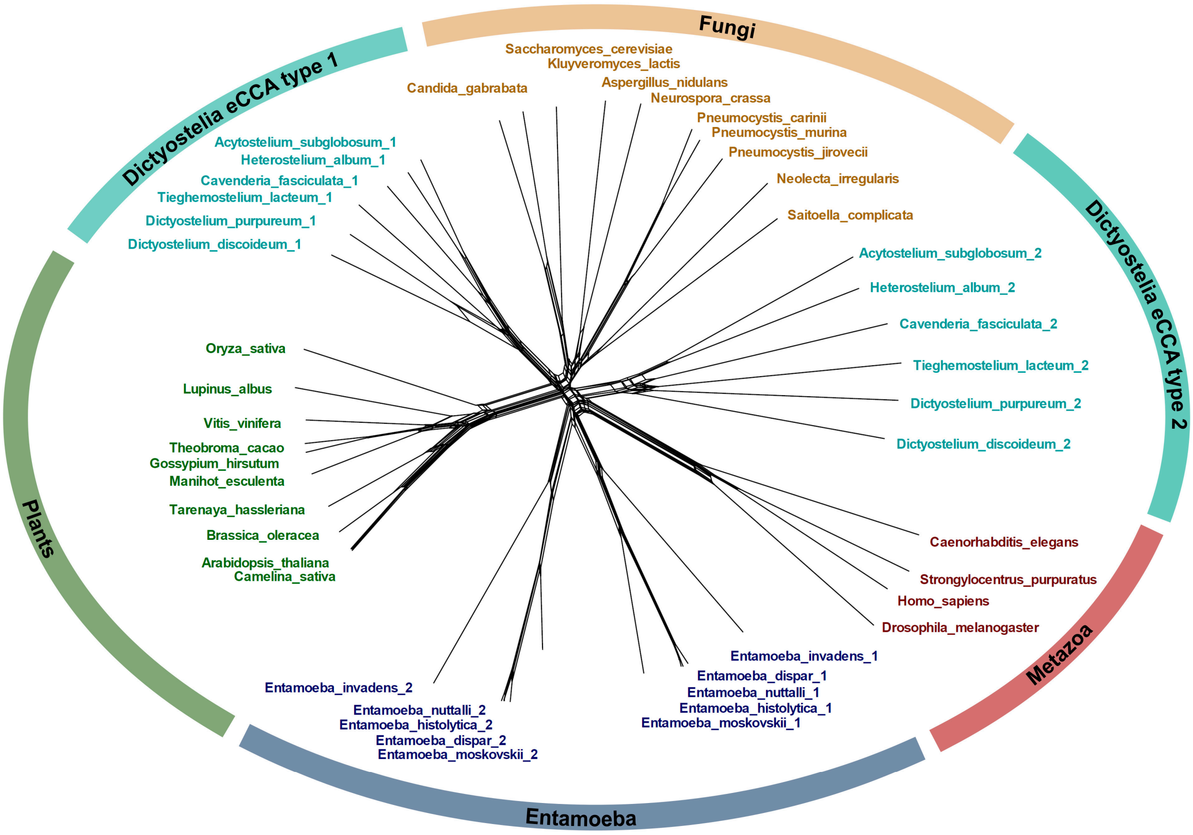

Figure S1. Phylogenetic analysis of the two Dictyostelia CCA-adding enzyme types. Protein sequences of Dictyostelia, Fungi, Metazoa, Plants and Entamoeba CCA-adding enzymes were aligned using Clustal Omega [1] with standard settings. The alignment was used for phylogenetic tree generation using the program SplitsTree 4 [2]. The phylogenetic tree indicates that both types of Dictyostelia CCA-adding enzymes are only distantly related because they do not share a common branch, but have separate splits. The Dictyostelia eCCA1 (ancestral eukaryotic CCA-adding enzyme type 1) enzymes branch more closely with Plants and Fungi enzymes, whereas enzymes of type 2 (eCCA2) branch more closely to the metazoan enzymes.

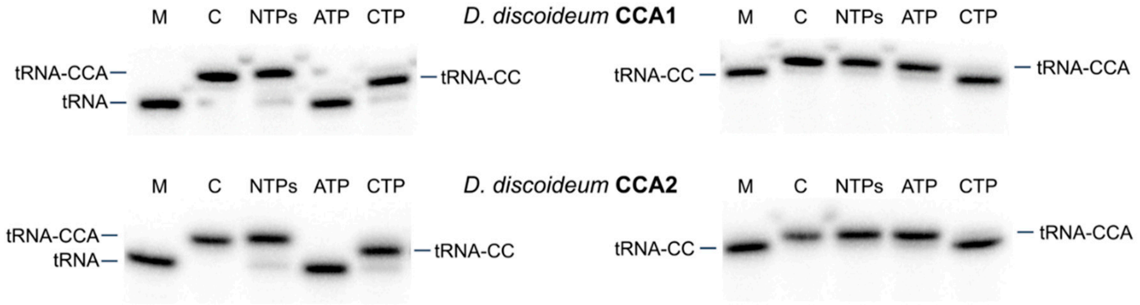

Figure S2. Both CCA1 and CCA2 synthesize full CCA-ends. Recombinantly expressed CCA-adding enzymes of *D. discoideum* were incubated with a α-^{32}P-labeleld tRNA substrate that lacks the CCA-terminus (tRNA) and a tRNA substrate that carries a CC-end, where only the terminal A residue is missing (tRNA-CC). Left panel: In the presence of only CTP, both enzymes add two residues to the tRNA lacking the CCA-end, resulting in tRNA-CC. In contrast, ATP is not incorporated at all. The same result is observed in the presence of all four nucleotides (NTPs). Right panel: On tRNA-CC, both enzymes add a terminal A residue when ATP (or NTPs) is offered. These results proof that CCA1 as well as CCA2 are bona fide CCA-adding enzymes and do not represent tRNA nucleotidyltransferases with partial activities as found in some other organisms [3–7].
In these assays, yeast tRNA^{Phe} was used as substrate. As the structure of this *in vitro* transcript is highly similar to that of the native and fully modified tRNA [8], it is frequently used as a standard substrate for the investigation of *in vitro* tRNA processing events [9–13]. M – mock incubation of labeled tRNA transcripts without enzyme. C, migration control consisting of labeled tRNA carrying a CCA-end.

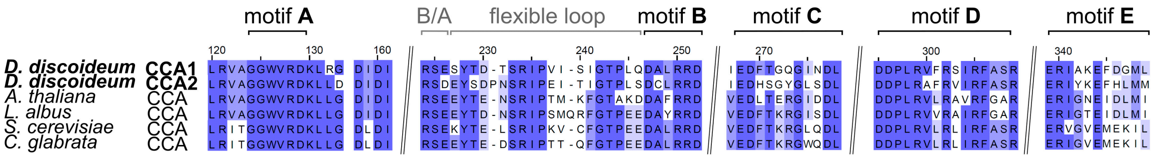

Figure S3. Sequence alignment of the two *D. discoideum* CCA-adding enzymes with eukaryotic CCA-adding enzymes with known CCA-adding activity. The two protein sequences of *D. discoideum* CCA-adding enzymes were aligned with protein sequences of the eCCA enzymes of *A. thaliana* (AC: Q94K06), *L. albus* (AC: Q42867), *S. cerevisiae* (AC: P21269) and *C. glabrata* (AC: Q9P4S5) using Clustal Omega [1] with standard parameters. All aligned protein sequences share the same conservation of the core motifs A – E, the basic/acidic motif and the flexible loop element. These motifs characterize the *D. discoideum* CCA-adding enzymes as ancestral eukaryotic type enzyme (eCCA).

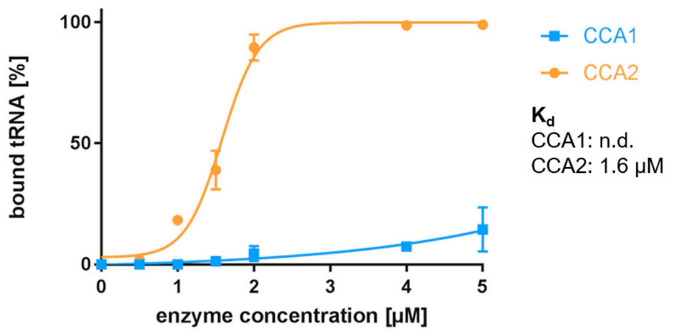

Figure S4. Interaction of CCA1 and CCA2 with tRNA-CC is not affected by ATP. Gel shifts were performed after preincubation of enzymes with 1mM ATPγS. While tRNA binding of CCA2 shows no significant difference to the interaction without ATPγS (Fig. 5 in the main text), CCA1 shows almost no interaction, as seen in the absence of ATPγS.

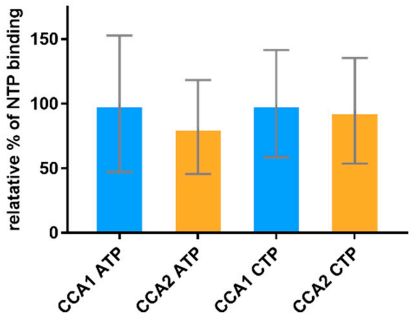

Figure S5. CTP and ATP binding behavior of CCA1 and CCA2. Nucleotide binding of CCA1 was set to 100%. The binding behavior of CCA2 is shown relative to CCA2, indicating only marginal deviations. NTP interaction was quantified in a comparative nucleotide binding assay according to Lizano *et al*. [14].

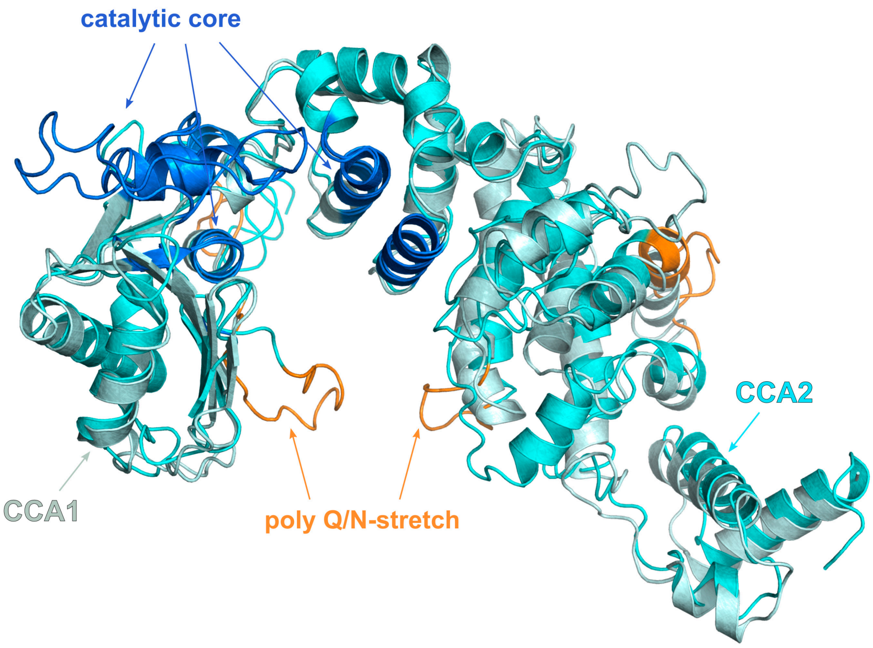

Figure S6. Structure prediction of the two *D. discoideum* CCA-adding enzymes. The structures of the CCA-adding enzymes were calculated using iTasser [15]. The core motifs of the two enzymes are colored in blue. The CCA-adding enzyme CCA2 (cyan) harbors several Q/N stretches (orange) that are not found in CCA1 (grey).

Accession numbers of the protein sequences used in Figure S1:

	Species	**Accession number**
**Metazoa**	*Caenorhabditis elegans*	Q93795
	Drosophila melanogaster	AAF51964
	Homo sapiens	BAB70662
	Strongylocentrotus purpuratus	XP_784774.4
**Fungi**	*Aspergillus nidulans*	KZN89459
	Candida glabrata	Q9P4S5
	Kluyveromyces lactis	Q6CSU9
	Neolecta irregularis	(prospilgn)
	Neurospora crassa	Q7SAL9
	Pneumocystis jirovecii	XP_018230996.1
	Pneumocystis carinii	XP_018225359.1
	Pneumocystis murina	XP_007874977.1
	Saccharomyces	P21269
	Saitoella complicata	XP_019025108.1
**Plants**	*Arabidopsis thaliana*	Q94K06
	Brassica oleracea	XP_013637841.1
	Camelina sativa	XP_010477526.1
	Gossypium hirsutum	XP_016698247.1
	Lupinus albus	Q42867
	Manihot esculenta	XP_021601417.1
	Oryza sativa subsp japonica	Q2QX33
	Tarenaya hassleriana	XP_010534110.1
	Theobroma cacao	XP_007020008.2
	Vitis vinifera	A7QGJ5
**Entamoeba**	*Entamoeba dispar* 1	XP_001739538.1
	Entamoeba dispar 2	XP_001740657.1
	Entamoeba histolytica 1	XP_652076.2
	Entamoeba histolytica 2	XP_654783.1
	Entamoeba invadens 1	XP_004183347.1
	Entamoeba invadens 2	XP_004257247.1
	Entamoeba moskovskii 1	EMO_031410-1
	Entamoeba moskovskii 2	EMO_113580-1
	Entamoeba nuttalli 1	XP_008860114.1
	Entamoeba nuttalli 2	XP_008854811.1
**Dictyostelia**	*Acytostelium subglobosum* 1	XP_012758000.1
	Acytostelium subglobosum 2	XP_012749843.1
	Cavenderia fasciculata 1	XP_004361592.1
	Cavenderia fasciculata 2	XP_004359300.1
	Dictyostelium discoideum 1	DDB_G0293504
	Dictyostelium discoideum 2	DDB_G0271378
	Dictyostelium purpureum 1	XP_003291342.1
	Dictyostelium purpureum 2	XP_003283778.1
	Heterostelium album 1	XP_020429327.1
	Heterostelium album 2	XP_020431701.1
	Tieghemostelium lacteum 1	KYQ91040.1
	Tieghemostelium lacteum 2	KYQ90023.1

Sequences of the Dictyostelia tRNA nucleotidyltransferases used in our analysis

>Acytostelium subglobosum 1
MEHSDKKIKTKHIPITLISNDYKHDNKFFHKSCTKTQAQATSTPTLTQQVNTPTPTTPTPDTKCNDVEVNNNNNN
NITNDIDSKNRRRDYVDAAVGNTGETVPLYISETERRLFEVLLQVVEKSKCGTTLRVAGGWVRDKLRGDDSSDID
IALDNLMGEAFASLVNQYLQDHNQETHRIGVIQSNPSQSKHLETATVRIFDMWIDFVNLRSETYTEESRIPTIQI
GTPQEDAFRRDLTINSLFYNINENKIEDFTGNGINDLMCGIVRTPLPPQTTFLDDPLRVLRSIRFATRLYYAIDH
KLVEAASSPKVKEALKSKVSHERVGIELEGMLSGPRPDLAIHLIHDLGLFDHIFSLPEGVTIKEKNYRFIATENI
LNMMRFINWGVANEEVPTRKLRLVSSLLQVFKDYNYTTKKNRQLPLIQHLMVEFLKFSNKDYDDVLNVLECAEKF
KQHVANLDIKGTFSRKDVGLIMYKAGPLWRTALANSLISELPKYNRNLSYPLHKPELIDGVPSPSSPMHDFHLHS
HNPHAHHPPLCEASKAIVAKYDNFVASVASHDLIGVWNIKKHLDGKMVMEMLNMKPGKWLAGVLQEIFEWQLDNP
KLDQEHCKQWVLNKFSSLPKQ

>Acytostelium subglobosum 2
MFVLRPGHWHQLQQQHNSIRSIRSINSSSINSIINHRQYTTTTTTTRKTKTKTAQTEPRLLVPQQQELDASSAIV
VSRLSNREKQFFDMFKSVASHYGGNIHLRVSGGWVRDKLLTTTSANPKKVDDIDIAIDGITGREFVDLIERYRVE
KGLSQQKYLIRSNPEKSKHLETAAIKIDGCTFDFTGLRAEDYDQHSRIPKISAGTLESDAHRRDITINALYFNLN
TYMVEDYTGKGLDDLRKGIVRTPLEPMITLLEDPLRALRVIRFATRFNFQIHPALYKSLTSSDVRKSLELKVSKE
RIYVEFTSMMSSFKSGAIQYYRYLVGTGLVDAVFPFDSMVKQPVEMIPRHLEKEIILSSLLLPLYIKYGKSDNIN
MEQLLMDFKLPKKEIRAVLSIIQSQEIIAKSVSSMLMISPDTLYHPDQFETSLRPLLEWLRHNQYWQAGLWLSSV
HFKLKFHSPDFNKDQFWIEFEKLIQNSLEKMANQQYCLDGHEIKDALSIPQEESKPITNIIDELLIWQWKYNICH
DKITKDDAKHFVRGLWAQQQLPHQ

>Cavenderia fasciculata 1
MFEKGSSSLSPLSSSSTTIVYNNNHKHLIEKEESQQEFKKRMKTKVIPITQITKDQQSYNPKYYHSVDLEAIDKD
TKEFYNVVVAIGDKDSTDTATIEITKVEQKLFQILLDVAKVNNCNTTLRVAGGWVRDKLRGDNSHDIDIALDNMM
GEAFAALVNKYLEDHHQETHRIGVIQSNPDQSKHLETATVKIFDSWVDLVNLRSETYSSNSRIPEIKIGTPEQDA
FRRDLTINSLFYNVNENRIEDFTGNGIVDLKCGIVRTPLPPLTTFLDDPLRVLRSIRFATRLYYAIDHKLVEAAS
NPTVKEALKSKVSHERIGIELDGMLNGPRPDLAIQLIYEFGLFDHVINIPDSIEIQDKEYKYKSILNVLNMMRFI
NWGTQAEEASTRKTRLLASLCANIYGYTFKNPLKKNKDTPILQHVLVDYLKVSNKECDEITNVLECAKLFRPLVN
QFYTNHVFSRKSVGLVIYKAGPFWKSSLAIALISELPEFQHNLVYPLHKEEINNSPSHDFYLHLSHPNHHHPPLC
DESKDIEIKYDQFCATVANHDLVGVWNIKKLLNGKQVMDMLQRKPGEWLTPLLQDVFEWQLDNPQLGEKECKEYV
LSKFSPK

>Cavenderia fasciculata 2
MYLNRIGSFCINSSKRLNFSTGTITSSTTTSATTSSGSPFFIIPPSSSSTSSTSSTSSPPSEFRKITNPLKSRLN
ASTIVMQPTTSLIFPMLFKHLSPQLERVGNPYKILDLTADEKYYFNLFRDVLIYFKRQDVQLRVAGGWVRNKLLG
IQGGTDIDIAIDNLTGHEFVQMIQKYYSINNQILFRNYIIKSNPEKSKHLETASLVMKDGLTFDFTGLRSEEYDE
FSRIPNIVKGTLISDSHRRDITINSLYYNLMTTRVEDYTLMGLPDLENKIVRTPLSPTLTLCEDPLRALRVLRFS
NRFSFSIESQLYESLCSDRVRDLLGLKVSRERVHTEFTSMMMSYKHAIPYFKTLVDTGLIHSIFPTQGLITTQDW
YQSLHYLEQSQHVSNIVPDQSIQEFILSSIFLPLKLKYQNHLDVFKILLDLKYSKKICFNVSSTLLSYEILKDPI
QLITSRPLEHLIDDNQLFEGSVLPLLKWLRHNQLWKNSLWTARLYFYVNHHPVLSRDDQIWIDAQQIIEKEIMPI
SNTKFCFEARAVMDALGIVNSCDIRPAIDQIMVYQWKNKSTSFSLDHIVKNQFFKIT

>Dictyostelium discoideum 1
MYTRPISKIEKSQDGYDRLYFHKQTNNEKEKLSPMQTSTPTSLKKDQPHENIKVSIYYSNERESESVDIRLTECE
IQLFKELMHVIRDSGCGTTLRVAGGWVRDKLRGDDSNDIDITLDNMMGEAFAELVNKHLSSKHHQTHRIGVIQSN
PEQSKHLETATVKVFDMWIDFVNLRSESYTDTSRIPVISIGTPLQDALRRDLTINSLFFNINENRIEDFTGQGIN
DLRDGIIRTPLPSLTTFLDDPLRVFRSIRFASRLNFKIDEELFKAGSDPVVKDAIKSKISHERIAKEFDGMLSAE
EPYLSIELINKFGLFDCLFSLPTVGLDETYIEQLKNSNKDSEKYCEIADSWIGMGKQYEEIDTKRQLILSALMIP
FYGIKFKNANKKNKEMSIIHYMFIEYIKFSNKDYDDVSLILECSEQLMEHIIKFVSAGIFNRKEIGLIIHKSAQL
WIVVIIISFIKIQYRPNKLQTLFSRYDKPENEKYTIFHKFVGEIKEHDLIGIWNIKRLLNGKQVQDLLNRKPGTW
LAP
VIQLILEWQLENPSKTEQDCKDWLLTLDIK

>Dictyostelium discoideum 2
MEILSAFQKTAKIFNNNNNNNNSYYLNKLFYSNNSIPTKNIQISPEENKLFDFLKNILIFNNRNDIELRVAGGWV
RDKLLDNQKQQQPQPQPQPQQQQQIKDIDIDIDIALSNISGTNFIKLIEEFKINKESFKKYLIKRNPEKSKHLET
ASINIHGFQIDFNSLRSDEYSDPNSRIPEITIGTPLSDCLRRDLTINSLFYNLNTLKIEDHSGYGLSDLENSIIR
TPLEPLKTLLDDPLRAFRVIRFASRFQGFQIEQQLYNTIKFNLNKQLISNKVSKERIYKEFHLMMSNKSSVIQSI

DYLIDTGLINSIIDFDKIINDHEIIKELFKKSLKYLKILINQNNNNNDNLFNIDFYKCCIFLPFFIDAHLGFKDN
TSKTIKSLINYQFSTNTGSTLNISLLFNNLISSFLNINNDTNNNTNNNTNNINQLKDFKLYLNNLNDKSNLYSIF
HELKNCKAGGLQWNFPLNISIVYLLEKYPDRENEILDVLNQLTKLFSDDNFTFEKVSLLVDGRKIKNLFPDQSID
ITNLMKSMTILQYENPNFKEDDILKWINKNIKDFILKI

>Dictyostelium purpureum 1
MDNVVNSEKKLKNKLAIPITTIKQDHNNYDKCYFHKASSTTENEIDNDYFKINVGIDSNNGEIKSTEIHLTKSEV
LLFQELMHVVRNSNCGTTLRVAGGWVRDKLRGDDSNDIDITLDNMMGESFAELVNKHLSGKQHQTHRIGVIQSNP
EQSKHLETATVKIFDLWIDFVNLRSETYTDNSRIPEIKIGTPLEDSLRRDLTINSLFYNINENKIEDFTGKAITD
LNLGIIRTPLPPLTTFLDDPLRVFRSIRFASRLYFKIDQELVEAASNAVVKEAIKSKISHERIAKEFDGMLGGKR
PDLAISLIYRFGIYDCLFSLPTSGLDHTKVHTEYREQSIYYCSQAMRLIEWGSQDEELSTKRQRLLSALCIPFYG
ITYLNANKKNKETSVIHYMIIEYLKLSNKDNDEISLVLDTAYQIRPHVLKYKEEGPSSFSRKNVGLIIHKCGVLW
RAALAIALILDLPKYNFNHFNFPTLCDESNLIINHYNLFCDEIKEQDLIGIWKIKRMLNGKQVQDLLNRKPGEWL
APVVQSILEWQLENPTLNENDCKKWLLETFK

>Dictyostelium purpureum 2
MNQLLNNLKKIKINNLFKKTKKNIDFEKDLNENEKHIIGYLKQVLQYHNRNDIELRVAGGWVRDKLLNYKNNKLD
LDFAINNTTGVEFVEMIQKYRTEKELKTVYSEYTIKMNPEKSKHLETVSITIDDFTIEFNALRSDFYDENSRIPQ
IQVGTLLSDSLRRDLTINALFYNINTLMVEDHSGYGLADLENGIIRTPLEPFNTLTEDPLRAFRILRFATKFNFK
IEPLLYQHIKTINNNIIKKVSKERIYTEFFLMMSVREYSEQYFSYLLDTGIINSIFIVNDYIPSSATLEQSVEIW
KDSLEFIKTSNNLFPNTKEIENLDFYITLIFYPLYKNCKLKKTEIISVLKEFKSPSKLIKQMERSFEVSELFSIL
ILKYINQLKDSSNSNRDQKSLKEFLKEEEQENEIYELIFSIKKYPDWRLSLYLSLVDLKYRINNNKIIIDGLAVS
NNSNNNIKLINLLEESLEINSNINDETNIAIKKLINNSNVNGWLNSVLDSFNELFESHCIPLSQVPFLMDGRELM
DLFPSIKEKQAKDSRFLNQLIKTTYFYQLKNNIKTKQELINYLNKDFL

>Heterostelium album 1
MSEEDQQHNEKRMKTKHIPITLISSSNKHDNQFFHKKPHQSDATTNNTTSTTITTTTTTSDDNLDENNIVYTAIG
ESGETVPLNITKVEQQLFQTLLQVVEQTKCGTVLRVAGGWVRDKLRGDHSSDIDIALDNMMGEAFASLVNQYLKD
HKQETHRIGVIQSNPAQSKHLETATVRIYDMWIDFVNLRSETYSDHSRIPEITIGTPEQDAYRRDLTINSLFYNI
NENKIEDFTGNGVDDLKCGIVRTPLPPQTTFLDDPLRVLRSIRFATRLYYAIDHKLVEAASSSTVKDALASKVSH
ERVGIELDGMLSGPRPDLAIHLINHFGLFDHIFSLPEGVTIKEKNYQYIAAENILNMMRFISWGSTTEELPTRKI
RLLSSLLQVFHNYTFTTKKNRTIPAIQYTIVEFLKFSNKDYDDVLNVLECADRFKPEVQKLNDNGSFSRKDVGLI
VYKAGPLWRVALANALISELPKYNRSIVYPFQRLENPDGSPVPRSPMHDFHLHSHNPHVHHPPLCEDAKKIVSKY
DNFCASVASHNLVGIWNTKKLLDGKQVMELLKKKPGAWLAPVLQEVFEWQLENPNSNEEQCKQWVLNKFS

>Heterostelium album 2
MKKNLERARNILLEQKKTISQLQNQNQLLISNQNDIVEVVVVLTKTTTILNSNKIYNNNNNNNNSNHTKSKDDKV
IVEVSNRIISSLNDQEKRYFELFKSVAKHNNLQDKVELRVSGGWVRNKLLSIDSSSSGYFDIDIAISGITGREFV
DLIEHYRIEHGLSQKKYLVRRNPDKSKHLETASIVIDGYTFDFTGLRAEQYDTHTRIPQIVDGTPTTDAFRRDIT
INSLYFNLETYRVEDFTGNGIDDLCNKYIRTPLSPHITLIEDPLRALRVLRFATRFHFNIDPSLYRSIESIEVRS
ALGYKVSKERIKNEFSLMMSSYDSGAIQYFRYLVDTNLVDVVFQSTGSIQITKEDWLLSLNYLEHSLRFKHQLSK
EMQEDFILGVLMLPLKLKYHDALNLEYLLEHFKSSKKQVRTVLSIISSPEILINQVIKTLLLPSMTITKESLTQP
HLFDQITLPLLGWLRNNECWQMGLSISAVHSEIINLPIAHVDKGMN

>Tieghemostelium lacteum 1
MDNNNIKYIPITTIKEGQTFDKDYYQQKATPDTQSPKDGYYHVNVGVQVVDELKSATTIRVSEMEKKLFQLLMTV
VRESGCGSTLRVAGGWVRDKLRGDNSHDIDIALDNMMGATFAELVNKHLEETHQEVHRIGVIQSNPDQSKHLETA
TVKVYDMWIDFVNLRSETYTEGSRIPTIDIGTPLEDALRRDLTINSLFFNINENLIQDYIGTAIKDLQLGIIRTP
LPPLTTFLDDPLRVFRSIRFATRMYFAIDKYLMEAASNQQVKDAIHTKISHQRIAKEFDGMVTGPRPDLAIHLIQ
QLGLYQYLFALPPGYNELSKINYIDASCNYINQMMRLAHWGENSEELSTKRVRLLSALMIPFHTLTYKNPQNKNR
IQTLIQYILLDHLKLSNKDYDDVILILDCAKMFLPLITQFQTNNTFNRKLVGLAMAKAGVLWRASLSIAMVIELP
DHQFNFTFPMHDSEETPKIFNHHTHTHPHHQPMCDQSKNICLLYDQFCATIDTHGLIGCWSIKRLLDGNQVKEIL
QKAPGSWLAPVIQAIFEWQLENPTKTVDQCKEWLIATYK

>Tieghemostelium lacteum 2
MIKHSFKLLCKHIIITPNEQKLFQKLKNATVDRDDVTIRIAGGWVRNKLLAMKSEQKEWSIDEENKDIDIALDNI
NGSDFVNSIINIEKEPLRSYTVKLNPEKSKHLETASIVIDGYNIDCNGLRSDIYSQDSRIPLITKGTPLSDALRR
DITINTLFYNIHTQEIEDHTQQGLKDLSECLIRTPLSPFETLLEDPLRAFRVLRFATKFHFAIEPQLMGVIKSVD
FRKTVLKKVSRERINTEFNKLMTKPAYALQYFKYLVETGLVEIVYHHTPNPSLPFQNSLPYLELGNSQSVNTKVL
DTLEFRHSLIFYPMVLDNLLSLTDITKILRDFKSPNKEISQTLSILRLSQVLSTIKSHYTQDTQHTNTSIESYLI

```
DSPTNFKTHLFPLLLELRKSHQFLLSIHLCRIHSQINDNNNNNNSEQFWNDVEALISIKCYPLSKIKYSLTQEDV
FENVEIVKSGKCSGTGFTSLMNELIYHQFSKHNFKSNHIQPISKQEAIQFLNSLPNK
```

References

1. Sievers, F.; Wilm, A.; Dineen, D.; Gibson, T.J.; Karplus, K.; Li, W.; Lopez, R.; McWilliam, H.; Remmert, M.; Söding, J.; et al. Fast, scalable generation of high-quality protein multiple sequence alignments using Clustal Omega. *Mol. Syst. Biol.* **2011**, *7*, 539, doi:10.1038/msb.2011.75.
2. Huson, D.H.; Bryant, D. Application of phylogenetic networks in evolutionary studies. *Mol. Biol. Evol.* **2006**, *23*, 254–267, doi:10.1093/molbev/msj030.
3. Tomita, K.; Weiner, A.M. Collaboration between CC- and A-adding enzymes to build and repair the 3'-terminal CCA of tRNA in Aquifex aeolicus. *Science* **2001**, *294*, 1334–1336, doi:10.1126/science.1063816.
4. Bralley, P.; Chang, S.A.; Jones, G.H. A phylogeny of bacterial RNA nucleotidyltransferases: Bacillus halodurans contains two tRNA nucleotidyltransferases. *J. Bacteriol.* **2005**, *187*, 5927–5936, doi:10.1128/JB.187.17.5927-5936.2005.
5. Reid, N.E.; Ngou, J.S.; Joyce, P.B.M. Schizosaccharomyces pombe contains separate CC- and A-adding tRNA nucleotidyltransferases. *Biochem. Biophys. Res. Commun.* **2019**, *508*, 785–790, doi:10.1016/j.bbrc.2018.11.131.
6. Preston, M.A.; Porter, D.F.; Chen, F.; Buter, N.; Lapointe, C.P.; Keles, S.; Kimble, J.; Wickens, M. Unbiased screen of RNA tailing activities reveals a poly(UG) polymerase. *Nat. Methods* **2019**, *16*, 437–445, doi:10.1038/s41592-019-0370-6.
7. Erber, L.; Franz, P.; Betat, H.; Prohaska, S.J.; Mörl, M. Divergent Evolution of Eukaryotic CC- and A-Adding Enzymes. *IJMS* **2020**, *21*, 462, doi:10.3390/ijms21020462.
8. Shi, H.; Moore, P.B. The crystal structure of yeast phenylalanine tRNA at 1.93 A resolution: a classic structure revisited. *RNA* **2000**, *6*, 1091–1105, doi:10.1017/s1355838200000364.
9. Oommen, A.; Li, X.Q.; Gegenheimer, P. Cleavage specificity of chloroplast and nuclear tRNA 3'-processing nucleases. *Mol. Cell. Biol.* **1992**, *12*, 865–875, doi:10.1128/mcb.12.2.865.
10. Loria, A.; Pan, T. The 3' substrate determinants for the catalytic efficiency of the Bacillus subtilis RNase P holoenzyme suggest autolytic processing of the RNase P RNA in vivo. *RNA* **2000**, *6*, 1413–1422, doi:10.1017/s1355838200000959.
11. Hoffmeier, A.; Betat, H.; Bluschke, A.; Günther, R.; Junghanns, S.; Hofmann, H.-J.; Mörl, M. Unusual evolution of a catalytic core element in CCA-adding enzymes. *Nucleic Acids Res.* **2010**, *38*, 4436–4447, doi:10.1093/nar/gkq176.
12. Tretbar, S.; Neuenfeldt, A.; Betat, H.; Mörl, M. An inhibitory C-terminal region dictates the specificity of A-adding enzymes. *Proc. Natl. Acad. Sci. U. S. A.* **2011**, *108*, 21040–21045, doi:10.1073/pnas.1116117108.
13. Ernst, F.G.M.; Erber, L.; Sammler, J.; Jühling, F.; Betat, H.; Mörl, M. Cold adaptation of tRNA nucleotidyltransferases: A tradeoff in activity, stability and fidelity. *RNA Biol.* **2018**, *15*, 144–155, doi:10.1080/15476286.2017.1391445.
14. Lizano, E.; Schuster, J.; Müller, M.; Kelso, J.; Mörl, M. A splice variant of the human CCA-adding enzyme with modified activity. *Journal of Molecular Biology* **2007**, *366*, 1258–1265, doi:10.1016/j.jmb.2006.12.016.
15. Yang, J.; Zhang, Y. I-TASSER server: new development for protein structure and function predictions. *Nucleic Acids Res.* **2015**, *43*, W174-81, doi:10.1093/nar/gkv342.
